# Validation of Housekeeping Genes for Normalizing RNA Expression in Real-Time PCR in Tuberculomas and Peripheral Blood Mononuclear Cells for Pulmonary Tuberculosis Patients

**DOI:** 10.3390/ijms262211219

**Published:** 2025-11-20

**Authors:** Ekaterina K. Tarasova, Ekaterina N. Pavlova, Ekaterina Yu. Rybalkina, Ekaterina A. Scherbakova, Ruslan V. Tarasov, Maria V. Erokhina

**Affiliations:** 1 Central Tuberculosis Research Institute, 107564 Moscow, Russia; tarasova.ek.nano@yandex.ru (E.K.T.); kate_rybalkina@mail.ru (E.Y.R.); scherbakovakatya108@gmail.com (E.A.S.); etavnai@yandex.ru (R.V.T.); 2 Faculty of Biology, Lomonosov Moscow State University, 119234 Moscow, Russia; guchia@gmail.com

**Keywords:** housekeeping genes, pulmonary tuberculosis, validation of gene stability, *PPIA*, *YWHAZ*, *HPRT1*

## Abstract

Accurate normalization of qRT-PCR data in pulmonary tuberculosis (TB) research requires reference genes whose expression is invariant across clinically relevant matrices, yet no studies have addressed this in lesion tissue and blood concurrently. We assessed the expression stability of eight popular housekeeping genes—*ACTB*, *B2M*, *GAPDH*, *HPRT1*, *PPIA*, *RPL13A*, *UBC* and *YWHAZ*—in lung tuberculomas and peripheral blood mononuclear cells (PBMCs) from TB patients. Standardized extraction and amplification yielded Cq values that were ranked by geNorm, NormFinder, BestKeeper and comparative Delta CT, with consensus scores generated in RefFinder; and correlation analysis was conducted in order to select the most suitable genes to work collectively for future normalization. The consensus analysis placed *PPIA*, *YWHAZ* and *HPRT1* at the top, while *GAPDH* and *UBC* were the least stable. Our findings endorse a three-gene panel (*PPIA*, *YWHAZ*, *HPRT1*) for robust normalization of host gene-expression studies in both lesion tissue and PBMCs in pulmonary TB and highlight the necessity of context-specific reference-gene validation.

## 1. Introduction

Real-time PCR (qRT-PCR) is the most widely used method for quantifying gene expression in diverse clinical specimens obtained from patients with pulmonary tuberculosis—including sputum, surgical tissue, broncho-alveolar lavage fluid, whole blood and peripheral blood mononuclear cells [1,2,3,4]. This technique is pivotal not only for confirming the diagnosis but also for characterizing the tuberculous inflammatory response and its immunopathology, and for identifying molecular targets amenable to pharmacological intervention during the development of effective personalized host-directed therapies (HDT) [5,6,7,8].

Meaningful comparison of qRT-PCR data generated from different specimen types critically depends on the validation of reference genes, which are traditionally selected from the class of housekeeping genes (HKGs).

HKGs are typically described as being constitutively expressed across all cell types, tissues, stages of cell differentiation and the cell cycle, experimental conditions, and disease contexts. Consequently, normalizing RT-PCR output to appropriate HKGs is indispensable for accurate interpretation.

In clinical research—including studies of pulmonary TB—the genes *GAPDH*, *ACTB*, *B2M* or ribosomal RNA genes are most frequently chosen as reference genes [7,8,9]. However, recent studies have questioned their suitability as reference genes in human biospecimens [10,11,12,13], and analogous results have been reported in cultured cell lines [14]. These observations underscore the need to validate HKGs whenever clinical material is analyzed, including in pulmonary TB.

Publications that explicitly address HKG validation in pulmonary TB are scarce: one study assessed HKG stability in PBMCs [15] and another investigated total RNA from patients’ plasma [16]. No investigations have examined HKG stability in surgical lung tissue specimens or simultaneously across multiple specimen types from TB patients, highlighting the need for such work to prevent data misinterpretation.

Accordingly, the aim of this study was to evaluate and compare the stability of the most commonly employed HKGs in perilesional lung tissue samples and PBMCs from patients with pulmonary TB (tuberculomas). Expression stability was assessed with the RefFinder web tool, which integrates the algorithms NormFinder, BestKeeper, geNorm and Delta CT. We additionally performed correlation analysis to identify gene combinations suitable for multiplex normalization. We anticipate that these results will extend the current knowledge of HKG stability under the specific pathology of active pulmonary tuberculosis and will facilitate the selection of an optimal reference gene for qRT-PCR in both lung tissue and PBMCs, thereby improving the reliability of data supporting the development of HDT for this socially significant disease.

## 2. Results

### 2.1. Primer Validation

The performance of all primers (Table 1) was validated.

Amplification efficiencies for each gene were calculated from standard curves and ranged from 91.6 to 100.5%, with corresponding correlation coefficients (R^2^) from 0.996 to 0.999 (Table 2). For each primer set, a melting curve was obtained and a single peak was detected, confirming primer specificity (Figure S1a). Standard curves for the qRT-PCR are represented in Figure S1b and demonstrate slopes from 3.32 (for *PPIA*) to 3.54 (for *YWHAZ*) (Table 2).

### 2.2. Quantification Cycle (Cq) Stability Analysis of Genes in Groups “Tuberculomas” and “PBMCs”

**Figure 1:**
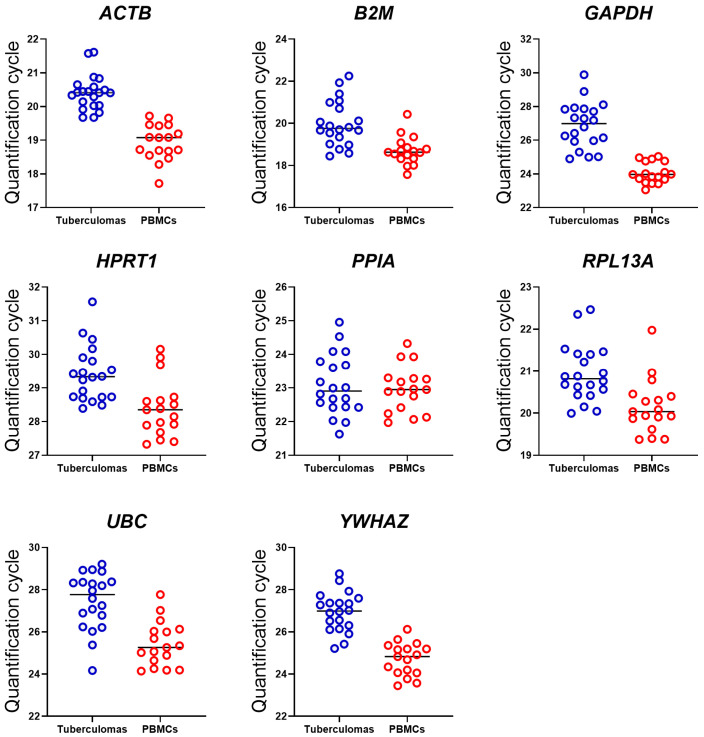
Swarm plots of Cq values obtained with means (shown as a black bar) for each gene from tuberculomas and PBMCs. All gene Cq_Mean_ values in PBMCs are lower than those in the “tuberculomas” group except for the *PPIA*. The Cq_Mean_ for *PPIA* remains the same in both sample groups, which is a feature unique to this gene.

To assess the expression stability of the selected genes in groups “tuberculomas” and “PBMCs”, we analyzed their expression profiles using descriptive statistics. In all specimens, every gene reached the detection threshold at similar Cq values; the mean Cq (Cq_Mean_) and its dispersion (swarm plot) for each gene are presented in Figure 1 and Table 3.

In both specimen groups—in “tuberculomas” and “PBMCs”—the gene requiring the highest number of amplification cycles was *HPRT1* (Cq_Mean_ = 29.42 and 28.40, respectively), whereas the lowest cycle number was observed for *B2M* (Cq_Mean_ = 20.04 and 18.70, respectively). The difference between their Cq_Mean_ is nearly 9.5–10 cycles.

The standard deviation (SD) of gene Cq values ranged from 0.5 to 1.44 in the “tuberculomas” group and from 0.53 to 1.05 in the “PBMCs” group. In the latter group, the SD for most genes lay between 0.6 and 0.8. Overall, the dispersion of SD values for Cq_Mean_ was greater in the “tuberculomas” group than in the “PBMCs” group. This finding indicates the greater stability of gene expression in PBMCs. Consistently, all gene Cq_Mean_ values in PBMCs were slightly lower than those in the “tuberculomas” group. The largest inter-group differences in Cq_Mean_ were observed for *GAPDH* (2.96 cycles) and *UBC* (2.12 cycles), whereas the smallest was for *PPIA* (0.11 cycles).

**Figure 2:**
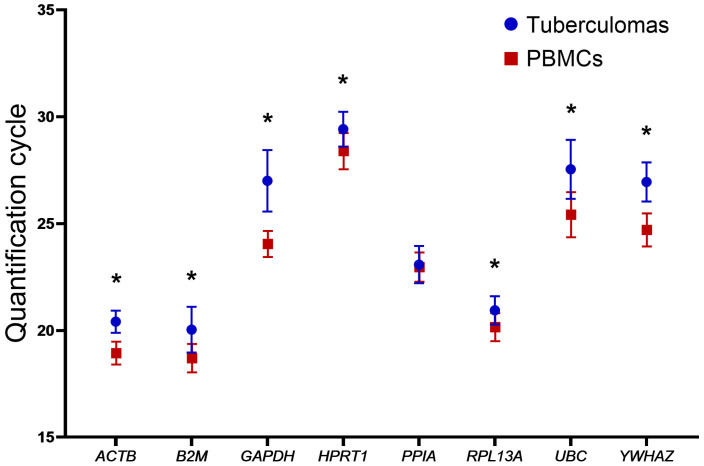
Comparison of the Cq_Mean_ values of the selected genes between samples from the two groups “tuberculomas” and “PBMCs”. Asterisks indicate significant differences. The values are represented as Cq_Mean_ (blue circles and red squares) ± SD (blue and red bars). All Cq_Mean_ values differ between the two groups, with the exception of the *PPIA* gene. For *PPIA*, the Cq_Mean_ shows no significant difference between the two sample groups.

All genes displayed statistically significant differences in Cq_Mean_ except *PPIA* (Figure 2 and Table 3).

The results indicate that gene-expression levels are comparable in both types of biomaterials: tuberculoma perilesional tissue and PBMCs.

Subsequent gene-stability analysis was performed with the most widely used statistical algorithms—geNorm, NormFinder, BestKeeper, Delta CT and RefFinder.

### 2.3. Ranking Gene-Expression Stability in Tuberculoma Tissue and PBMCs Using the geNorm, NormFinder, BestKeeper and Delta CT Algorithms

#### 2.3.1. Ranking Selected Housekeeping Genes Using the geNorm Analysis

**Figure 3:**
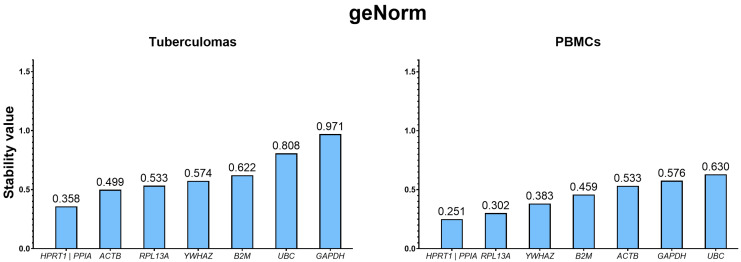
Stability ranking of candidate reference genes in the groups “tuberculomas” and “PBMCs” as determined by geNorm. Genes are ordered by their M value (lower M = higher stability), from the most to the least stable. For the group “tuberculomas”, the most stable genes are *HPRT1* and *PPIA*; the least stable are *GAPDH* and *UBC*, as well as for the “PBMCs” group. Figures are replotted from the original results obtained with the RefFinder web resource, and original figures are given in Supplementary Materials (Figures S2 and S7 for the “tuberculomas” and “PBMCs” groups, respectively).

geNorm ranks genes according to their M value—the arithmetic mean of expression variability across all analyzed gene pairs [12]. Gene expression is deemed stable when M < 1.5. By this criterion, the expression of all genes in both study groups qualifies as stable. However, the lowest M values—and thus the most stable genes—in the “tuberculomas” group were *PPIA* and *HPRT1* (both M = 0.358). The highest M values (least stable genes) were recorded for *UBC* (M = 0.808) and *GAPDH* (M = 0.971) (Figure 3).

In the “PBMCs” group, the greatest stability was likewise observed for *PPIA* and *HPRT1* (both M = 0.251). The least stable expression was found for *UBC* (M = 0.630) and *GAPDH* (M = 0.573).

Thus, geNorm produced similar gene-stability rankings for both specimen groups—“tuberculomas” and “PBMCs”.

#### 2.3.2. Ranking of Selected Housekeeping Genes Using the NormFinder Analysis

**Figure 4:**
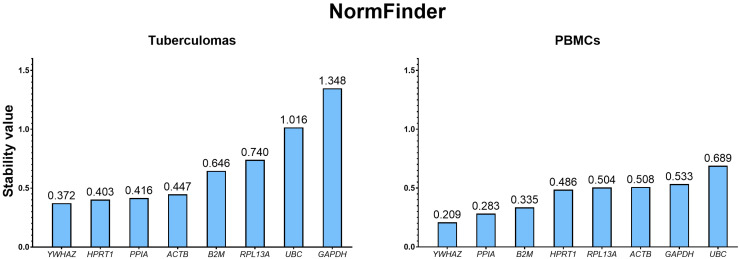
Stability ranking of candidate reference genes in groups “tuberculomas” and “PBMCs” calculated with NormFinder. Genes are ordered by increasing stability value (lower value = higher stability), from the most to the least stable. For both groups, the most stable gene is *YWHAZ*, while the least stable are *UBC* and *GAPDH*. Figures are replotted from the original results obtained with the RefFinder web resource, and original figures are given in Supplementary Materials (Figures S3 and S8 for the “tuberculomas” and “PBMCs” groups, respectively).

NormFinder employs a model-based variance-estimation approach [17] to rank genes by stability, whereby a lower value denotes greater stability. Using this algorithm, *YWHAZ* emerged as the most stable gene in both specimen groups—“tuberculomas” (0.372) and “PBMCs” (0.209)—whereas *UBC* and *GAPDH* were the least stable (Figure 4).

#### 2.3.3. Ranking of Selected Housekeeping Genes Using the Delta CT Analysis

The method calculates ΔCq values for every pair of genes and derives their SD [18]; a lower SD indicates greater expression stability.

**Figure 5:**
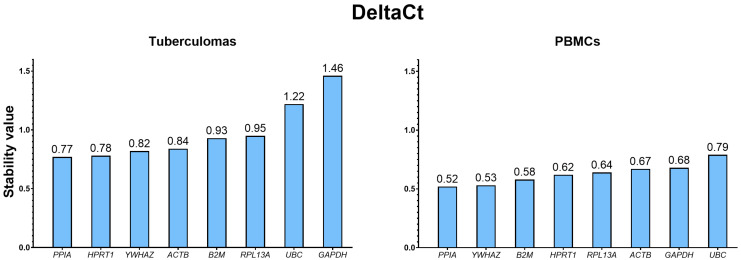
Stability ranking of candidate reference genes in groups “tuberculomas” and “PBMCs” calculated with the Delta CT method. Genes are ordered by increasing the Delta CT-derived stability value (lower value = higher stability), from the most to the least stable. For both groups, the most stable gene is *PPIA*, while *GAPDH* and *UBC* are the least stable. Figures are replotted from the original results obtained with the RefFinder web resource, and original figures are given in Supplementary Materials (Figures S4 and S9 for the “tuberculomas” and “PBMCs” groups, respectively).

In the “tuberculomas” group, the Delta CT algorithm identified *PPIA* (0.77) as the most stable gene, closely followed by *HPRT1* (0.78). The least stable genes were *GAPDH* (1.46) and *UBC* (1.22) (Figure 5).

In the “PBMCs” group, *PPIA* (0.52) showed the highest stability, with *YWHAZ* (0.53) ranking second. The least stable genes were *GAPDH* (0.68) and *UBC* (0.79).

#### 2.3.4. Ranking of Selected Housekeeping Genes Using the BestKeeper Analysis

BestKeeper ranks gene stability using the SD, the coefficient of variation (CV) of Cq values and the BestKeeper correlation coefficient (r) [19]. A low CV indicates little inter-sample variation in Cq and, therefore, stable gene expression. The BestKeeper correlation coefficient is computed as the correlation between the BestKeeper stability index (geometric mean Cq) and the Cq of each individual gene.

In the “tuberculomas” group, *UBC* and *GAPDH* were deemed unstable by the SD criterion because SD > 1 (Table 4; Table S1, Figure S5). By contrast, the CV criterion classified all genes as stable (CV < 5% Cq). Based on the correlation coefficient, the most stable gene was *YWHAZ* (r = 0.893) and the least stable was *GAPDH* (r = 0.513).

In the “PBMCs” group, all genes exhibited SD < 1 and CV < 5% Cq and were therefore regarded as stable by these metrics (Table 5; Table S2, Figure S10). According to the correlation coefficient, *YWHAZ* was the most stable gene (r = 0.961), whereas *GAPDH* was the least stable (r = 0.678).

#### 2.3.5. Integrated Ranking Based on RefFinder

**Figure 6:**
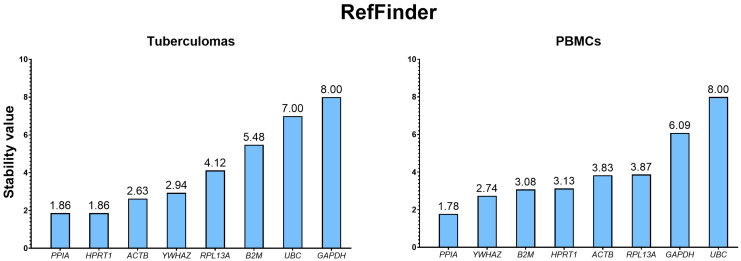
Integrated stability ranking of the analyzed genes in the groups “tuberculomas” and “PBMCs”, based on the geometric mean scores from RefFinder. The most stable gene in both groups is *PPIA*, while *UBC* and *GAPDH* are the least stable. Figures are replotted from the original results obtained with RefFinder web resource, and original figures are given in Supplementary Materials (Figures S6 and S11 for the “tuberculomas” and “PBMCs” groups, respectively).

Because the four algorithms apply different criteria, RefFinder [20] was used to calculate the geometric mean stability value for each gene across geNorm, NormFinder, BestKeeper and the Delta CT method. According to the composite ranking, *PPIA* is the most stably expressed gene in both groups, “tuberculomas” and “PBMCs” (Figure 6). The least stable genes are *GAPDH* and *UBC*. For optimal dual-gene normalization, RefFinder identified *PPIA*/*HPRT1* as the best pair in the group “tuberculomas” and *PPIA*/*YWHAZ* in the group “PBMCs”. Integrative tables with rankings from all algorithms are given in Table 6 and Table 7.

### 2.4. Correlation Analysis of Selected Housekeeping Genes in Groups “Tuberculomas” and “PBMCs”

To identify gene pairs suitable for joint use as reference controls, we performed a correlation analysis. We focused on correlations among *PPIA*, *HPRT1* and *YWHAZ*, the three genes ranked most stable by all algorithms described above.

**Figure 7:**
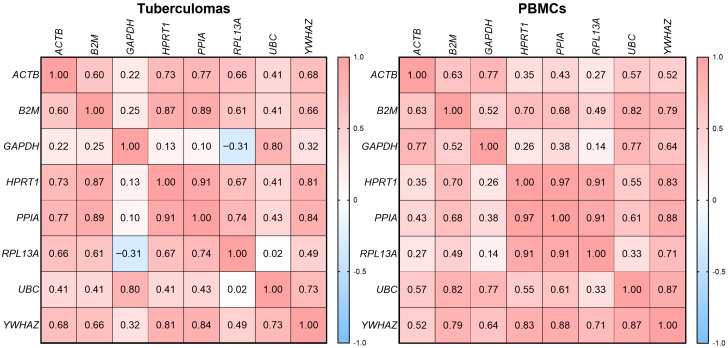
Heat map of Pearson correlation coefficients for the genes analyzed. Positive correlations are shown in red and negative correlations in blue. In both groups, the strongest correlation is observed between *YWHAZ*, *PPIA* and *HPRT1.*

Heat-map correlation matrices are shown in Figure 7. The strongest positive correlation in both specimen types was observed between *PPIA* and *HPRT1*: r = 0.91 (*p* = 9.0 × 10^−9^) in the “tuberculomas” group and r = 0.97 (*p* = 1.35 × 10^−7^) in “PBMCs”.

In the “tuberculomas” group, *YWHAZ* correlated strongly with *PPIA* (r = 0.84, *p* = 1.79 × 10^−4^) and *HPRT1* (r = 0.81, *p* = 7.0 × 10^−6^). In the “PBMCs” group, *YWHAZ* likewise showed strong positive correlations with *PPIA* (r = 0.88, *p* = 3.0 × 10^−6^) and *HPRT1* (r = 0.83, *p* = 3.0 × 10^−5^).

## 3. Discussion

We evaluated the stability of the most frequently used housekeeping genes in tuberculoma perilesional tissue and PBMC biospecimens from patients with pulmonary TB (tuberculomas). To identify the most stable HKGs for analyzing material from patients with pulmonary TB, we selected eight genes—*ACTB*, *B2M*, *GAPDH*, *HPRT1*, *PPIA*, *RPL13A*, *YWHAZ* and *UBC*—that are most commonly employed as reference genes in clinical studies [11,15].

Historically, *ACTB*, *GAPDH*, *HPRT*, and 18S were regarded as universal reference genes. They were widely used to normalize signals in Northern blots, where only a qualitative assessment of expression was needed [21]. With the advent of quantitative PCR, the use of these genes required re-evaluation.

The need to refine and validate reference-gene selection also stems from their potentially unstable expression, which can depend on experimental conditions. In particular, regulatory variability in the expression of these housekeeping genes was demonstrated long before qPCR emerged. Several studies report the variable expression of housekeeping genes such as 18S [22], *GAPDH* [23], *HPRT1* [24] and *ACTB* [25], both across tissue types and under pathological conditions. More recent evidence likewise points to instability in the expression of traditional housekeeping genes [26,27]. These findings have called into question the continued use of traditional housekeeping genes and have triggered a search for new reference genes that are better suited to specific experimental contexts [28,29].

In 2009, the journal Clinical Chemistry published the first Minimum Information for Publication of Quantitative Real-Time PCR Experiments guidelines (MIQE) [30], which established as a gold standard the prior validation of reference-gene stability and the use of at least two reference genes in any study. In 2025, these guidelines were further expanded and updated [31].

Nevertheless, many contemporary studies continue to deploy a single “classical” HKG without verifying its stability. Specifically, a systematic review on reference-gene selection for qPCR experiments notes that only 19 of 128 screened studies analyzing gene expression in vertebrates validated the stability of their reference-gene panel under the given experimental conditions. Only a small fraction of publications provide a rationale for reference-gene choice that takes the specifics of the experiment into account [10]. Therefore, our comprehensive analysis of eight commonly used candidate genes in tuberculoma samples and peripheral mononuclear cells demonstrates for the first time which housekeeping genes maintain stable expression in pulmonary tuberculosis across different clinical samples. Consequently, our study substantially broadens the current understanding of how to select reliable housekeeping genes for quantitative qPCR in this pathology.

We analyzed the Cq values of the selected genes in two clinical specimen types—tuberculoma perilesional tissue and peripheral blood mononuclear cells. All eight genes were reliably detected in both sample groups; however, lower Cq values were recorded for PBMCs, indicating a higher baseline transcript abundance. These differences appear to reflect morphological and cellular characteristics of the material: PBMCs constitute a relatively homogeneous cell population, whereas tuberculoma perilesional tissue is heterogeneous in cellular composition and exhibits fibrotic changes. Taken together, these factors lead to higher Cq readings. The greater homogeneity of PBMCs is also evident in their smaller Cq standard deviations, i.e., greater within-group expression stability relative to tuberculoma perilesional tissue.

In tuberculoma perilesional tissue, two expression tiers were observed: *ACTB*, *B2M*, *PPIA* and *RPL13A* amplified at Cq < 25, whereas *GAPDH*, *HPRT1*, *UBC* and *YWHAZ* amplified at Cq > 25. The lowest Cq was recorded for *B2M* (Cq ≈ 20.04), and the highest for *HPRT1* (Cq ≈ 29.4). A similar pattern was evident in PBMCs: *ACTB*, *B2M*, *PPIA* and *RPL13A* reached the threshold between cycles 18 and 23, whereas *GAPDH*, *HPRT1*, *UBC* and *YWHAZ* did so after cycle 24. This distribution underscores differences in baseline transcription of the selected housekeeping genes and permits their provisional classification as “highly” or “weakly” expressed in both clinical specimen types.

The greatest disparity is observed for *GAPDH*: lower Cq values in PBMCs indicate a markedly higher abundance of its transcripts in mononuclear cells than in tuberculoma perilesional tissue. This finding suggests that the gene’s expression level is strongly influenced by both the cellular composition of the sample and the metabolic activity of the cells during inflammation [32].

The smallest divergence in mean Cq values was recorded for *PPIA*, indicating comparable expression of this gene in both tuberculoma tissue and peripheral blood mononuclear cells.

The observed differences could be mitigated by varying the amount of input material added to the reaction. However, we deliberately adhered to a single sample-preparation protocol to eliminate additional variation associated with differing sample-handling methods.

To identify the most stably expressed housekeeping genes, we employed four established algorithms—geNorm, NormFinder, Delta CT and BestKeeper—and calculated the overall stability ranking with the RefFinder web tool. This multi-algorithm strategy is in line with current recommendations.

According to geNorm, the most stable genes were *PPIA* and *HPRT1* in both tuberculomas and mononuclear cells, whereas *UBC* and *GAPDH* were the least stable.

Analysis with the NormFinder algorithm confirmed the trends identified by geNorm. In the “tuberculomas” group, *YWHAZ* displayed the most stable expression; *HPRT1* showed nearly identical stability, whereas *PPIA* ranked third with a small gap. In the “PBMCs” group, *YWHAZ* again led the ranking, followed by *PPIA*, while *HPRT1* dropped to fourth place in this group. *UBC* and *GAPDH* exhibited the least stable expression in both groups of samples, consistent with the geNorm findings.

The Delta CT algorithm yielded results similar to the preceding algorithms. This overall agrees with the results produced by the other algorithms. In full agreement with the other algorithms, Delta CT classifies *UBC* and *GAPDH* as the least stably expressed genes.

The greatest divergence from the conclusions of the other methods was produced by the BestKeeper algorithm, owing to its composite assessment of stability. The literature emphasizes that r should be treated as the principal criterion, because SD and CV largely capture technical fluctuations arising from the experimental protocol [33]. Under this view, *PPIA* ranks first or second in stability in both tuberculoma tissue and peripheral mononuclear cells, fully consistent with the outputs of geNorm, NormFinder, and Delta CT. Giving priority to SD and CV, however, shifts the lead to *ACTB*, while *PPIA* falls into the mid-range of the ranking. Thus, the discrepancies displayed by BestKeeper reflect methodological rather than biological differences. Importantly, regardless of the chosen metric, this algorithm—like the others—consistently classifies *GAPDH* (and *UBC* as well) among the least stable genes.

An inversion is observed for the pair of least stable genes: in the “tuberculomas” group of samples, the least stable gene is *UBC*, with *GAPDH* ranking next, whereas in the “PBMCs” group the order is reversed—*GAPDH* becomes the least stable and *UBC* the second least stable. These observations underscore that the robustness of “classical” reference genes depends not only on pathophysiological context but also on the specific type of clinical material, reaffirming the need for prior validation of the reference-gene panel for each experimental model.

A subset of genes displays an intermediate level of expression stability, with performance dependent on the specimen type and the algorithm applied. In the “tuberculomas” group, *ACTB* proved to be a reasonably reliable reference gene, whereas in the “PBMCs” group, its expression was more variable. Across all methods, *RPL13A* consistently occupied the middle of the ranking without sharp drops; it can therefore serve as an auxiliary control gene but does not provide optimal normalization on its own. *B2M* showed moderately stable expression; however, its transcription is known to increase during immune activation [34] and tuberculosis [35], warranting caution when using this gene. *YWHAZ* ranked highly in NormFinder and Delta CT but dropped lower in geNorm, indicating higher intragroup variability and making it more dependable when paired with another stable control rather than as a sole reference. Overall, these “middle-tier” genes can strengthen a normalization panel if combined with one of the highly stable leaders and validated under the specific experimental conditions.

The integrated analysis performed with the RefFinder web platform confirmed and refined the conclusions of the individual algorithms. By combining the rankings from geNorm, NormFinder, Delta CT and BestKeeper through geometric averaging of their ranks, RefFinder provides the most balanced assessment of stability. As a result, *PPIA* occupied the top position in both tuberculoma perilesional tissue and mononuclear cells, while *YWHAZ* and *HPRT1* consistently rounded out the top three. The concordance between the RefFinder ranking and the outputs of the individual algorithms attests to the high consistency of the data obtained and underscores the biological robustness of these genes’ expression under conditions of active tuberculous inflammation. At the same time, the tool maintained low stability ranks for *GAPDH* and *UBC*, further confirming their limited suitability as single reference genes. Thus, the comprehensive RefFinder approach not only validates the selection of the optimal panel (*PPIA*, *YWHAZ*, *HPRT1*) but also demonstrates the practical necessity of employing multiple independent algorithms when finalizing the most stable reference genes.

Accordingly, the integrated analysis with RefFinder—synthesizing the outputs of geNorm, NormFinder, Delta CT and BestKeeper—showed that *PPIA*, *YWHAZ* and *HPRT1* display the highest expression stability and can therefore be recommended as the optimal reference panel for both tuberculoma perilesional tissue and peripheral blood mononuclear cells.

Correlation analysis of Cq values revealed the strongest correlation between the genes *PPIA* and *HPRT1*. The tight coupling of these transcripts indicates co-regulation and confirms that using them as a pair affords the most stable expression ratio across different specimen types. *YWHAZ* likewise showed a high positive coefficient with *PPIA* and *HPRT1*, making it a reliable additional control, although a slight drop in correlation within the “tuberculomas” group points to greater biological variability. The weak correlation of *GAPDH* and *UBC* with the leading trio once again underscores their limited suitability. Thus, the selection of reference genes must be guided not only by individual stability but also by concordance of expression among the controls.

The final selection of the reference-gene pair in this study is guided not only by the ranking algorithms but also by current best-practice frameworks, including the EU-CardioRNA COST Action recommendations and the MIQE guidelines.

In 2022, the EU-CardioRNA Consortium published recommendations for the use of RT-PCR. The authors believe that these recommendations serve as a tool for clinical research, enabling the development of validated assays at the intermediate stages of biomarker research. They are applicable across all clinical research areas and help bridge the gap between research use only and in vitro diagnostics [36]. The recommendations emphasize the importance of the proper selection of multiple, stably expressed reference genes. They also provide Guidelines and a Checklist for the reference procedure for RT-PCR validation. These include primer validation and amplification efficiency (90–110%), both of which we report in our study.

The MIQE guidelines emphasize the importance of transcript abundance—the quantity of target RNA (or DNA) in the sample—stipulating that the expression level (Cq) of a reference gene must be comparable to that of the target transcripts. Consequently, evidence of expression stability alone is insufficient; the Cq values of the chosen control genes must lie within the same range as those of the genes under investigation.

Thus, the Cq difference between *HPRT1* and *PPIA* is 6.34 in tuberculoma perilesional tissue and 5.43 in PBMCs, corresponding to roughly a 64-fold disparity in quantity. Such divergence increases the likelihood of random pipetting errors, diminishes normalization accuracy [37], and may justify excluding *HPRT1* from the list of potential reference genes [38].

By contrast, for the *PPIA*–*YWHAZ* pair—which likewise exhibits high stability and a positive correlation—the Cq difference is only 3.87 cycles in tuberculoma perilesional tissue and 1.73 cycles in mononuclear cells, corresponding to no more than a ~15-fold difference in template quantity, four times lower than for the *HPRT1*–*PPIA* pair. The comparable abundance of both mRNAs—corroborated by other studies in which *PPIA* and *YWHAZ* are recommended as a reliable reference pair [29,39]—makes this combination the one that best meets MIQE criteria for normalization in both clinical specimen types, perilesional tissue and PBMCs. In addition, the stability of *PPIA* expression is specifically highlighted in the paper by Guaita-Céspedes et al. [40].

Thus, the *PPIA*–*YWHAZ* pair is the preferred normalizer for qPCR data in human tuberculoma perilesional tissue and peripheral blood mononuclear cells, because a ΔCq ≤ 4 between these genes minimizes systematic error and reduces the risk of misinterpretation, particularly when target transcripts amplify within a Cq range of 22–27. The *PPIA*–*HPRT1* combination remains an acceptable alternative, provided *HPRT1* is consistently detected at ≤30 Cq and the expression level of the genes of interest is comparable to both reference markers. In light of MIQE guidelines requiring comparable transcript abundance, *PPIA*–*YWHAZ* (ΔCq_Mean_ ≤ 4) should be regarded as the optimal reference set, whereas *PPIA*–*HPRT1* is permissible when its expression level approximates that of the target genes.

## 4. Materials and Methods

### 4.1. Patients

In the present study, to standardize sampling we used biospecimens obtained exclusively from patients diagnosed with tuberculoma, a clinico-anatomical form of tuberculosis defined by the Russian classification system [41]. The diagnosis was confirmed by radiological imaging, bacteriological assays (conventional microbiology and PCR), and a morphologist’s report based on histological sections of surgical tissue stained with hematoxylin–eosin and Ziehl–Neelsen. All patients received anti-tuberculosis therapy during the pre-operative period. Patients with comorbidities—diabetes mellitus or rheumatoid arthritis—were excluded from the analysis cohort. Surgical tissue specimens were collected from 21 patients (11 women and 10 men) aged 22–49 years between 2021 and 2022. Peripheral blood mononuclear cells were obtained from 17 patients (9 women and 8 men) aged 18–55 years immediately before elective surgery in 2024. All biospecimens were acquired at the Central Tuberculosis Research Institute (CTRI, Moscow, Russia).

Patient characteristics are summarized in Table 8.

All patient-involving procedures were approved by the CTRI Ethics Committee (protocol #1, 18 January 2021 and protocol #1, 22 January 2024). Written informed consent was obtained from every participant. No patient can be identified from any of the material presented in this manuscript.

### 4.2. Lung Tissue Sampling

Lung tissue specimens were collected during elective surgical procedures. The surgeon excised 2–3 cubic fragments (~3 mm × 3 mm × 3 mm each) from distinct regions of the perilesional zone, located ~0.5–1 cm from the tuberculoma wall. Further in the text this type of material is referred to as “tuberculomas group”. Samples were placed in microtubes (Eppendorf, Boston, MA, USA) pre-loaded with 2 mL ice-cold plain RPMI-1640 medium (Paneco, Moscow, Russia) and transported to the laboratory for homogenization within 5–10 min.

In the laboratory, samples were washed twice with ice-cold plain RPMI-1640 and then transferred individually to porcelain mortars. During grinding with a porcelain pestle, each specimen was flash-frozen in liquid nitrogen. Once a homogeneous consistency was achieved, 1 mL TRI Reagent (MRC, Beverly Hills, CA, USA) was added and the mixture incubated at room temperature for 10 min. The tissue was further homogenized in 1 mL TRI Reagent; 200 µL of the supernatant—avoiding tissue debris—was transferred to a 1.5 mL microtube (Eppendorf, USA). The TRI Reagent volume was adjusted to 600 µL. Samples were stored at −80 °C in an ultra-low-temperature freezer (Sanyo, Osaka, Japan) until RNA extraction.

### 4.3. Isolation of Peripheral Blood Mononuclear Cells

Eight to nine milliliters of blood were drawn from the patient’s antecubital vein after overnight fasting into citrate-dextrose anticoagulant tubes (ADC-A, Vacuette, Monroe, NC, USA). The blood was centrifuged at 1000× *g* for 15 min. The supernatant was discarded, and the cell pellet was resuspended to 15 mL with calcium- and magnesium-free Dulbecco’s phosphate-buffered saline (Paneco, Russia). The suspension was layered onto 8 mL Ficoll solution (Paneco, Russia; density 1.077 g cm^−3^) in a 50 mL tube and centrifuged at 400× *g* for 20 min. The turbid interphase ring containing mononuclear cells was collected into a 15 mL tube. The volume was adjusted to 15 mL with FACS buffer (Becton Dickinson, Franklin Lakes, NJ, USA). The mononuclear cells were centrifuged in FACS buffer at 800× *g* for 10 min. The supernatant was removed and erythrocyte lysis performed by adding 1 mL one-fold AbiLyse buffer containing ammonium chloride (Abisense, Sochi, Russia) to the pellet and incubating for 5 min at 25 °C. Following erythrocyte lysis, the cells were washed again by centrifugation in FACS buffer (800× *g*, 10 min). The supernatant was discarded, 1 mL TRI Reagent was added to the pellet, and the sample was frozen at −80 °C for storage. Samples were stored for no longer than 6 months before RNA extraction.

### 4.4. Total RNA Extraction, RNA Quantity, Purity and Integrity Analysis and cDNA Synthesis

For all specimens, RNA was extracted with TRI Reagent (MRC, USA) according to the manufacturer’s instructions. The aqueous RNA solution was stored at −80 °C without freeze–thaw cycles. Prior to analysis, RNA samples were assessed for quantity and purity using a NanoDrop 8000 spectrophotometer (Thermo Fisher Scientific, Waltham, MA, USA) and for integrity on a 1% agarose gel. Samples containing < 1 µg total RNA, displaying A260/280 ratios < 1.80, or lacking visible 18S and 28S RNA bands on electrophoresis were excluded from the study. The resulting gel–electrophoresis image is shown in Figure S12. Consequently, 21 surgical-tissue specimens and 17 PBMC samples were selected for downstream analysis.

For cDNA synthesis, 1 µg RNA was treated with DNase (Thermo Fisher Scientific, USA) following the manufacturer’s protocol. Reverse transcription (20 µL reaction volume) was carried out with the MMLV RT kit (Evrogen, Moscow, Russia) using 1 µL reverse transcriptase (100 U µL^−1^), 1 µL oligo (dT) primer (20 µM), 1 µL random decanucleotide primers (20 µM) and 0.5 µL RNase inhibitor (RiboCare, 40 U µL^−1^; Evrogen, Moscow, Russia). The mixture was incubated for 60 min at 37 °C, followed by 10 min at 70 °C, and then chilled on ice. After reverse transcription, 80 µL nuclease-free water was added to bring the final volume to 100 µL. The resulting cDNA was stored at −20 °C.

### 4.5. Quantitative Real-Time PCR

Real-time PCR was carried out with the commercial qPCRmix-HS SYBR Low-ROX master mix (Evrogen, Moscow, Russia), which contains polymerase, buffer and dNTPs, following the manufacturer’s protocol. Each reaction contained 250 ng synthesized cDNA and primers at a final concentration of 500 nM in a total volume of 25 µL. qPCR was performed on a 96-well QuantStudio 12K Flex thermocycler (Applied Biosystems, Waltham, MA, USA).

### 4.6. Primer Characteristics

Primers were synthesized by Evrogen, Russia. Primer sequences are listed in Table 1.

Each sample was amplified in two technical replicates using the following qPCR cycling program: 95 °C for 3 min, then 40 cycles of 95 °C for 15 s and 60 °C for 60 s. Primer specificity was verified by melting-curve analysis. During the final amplification cycle, the following temperature profile was applied: 95 °C for 15 s (denaturation), 60 °C for 1 min (annealing) and 95 °C for 15 s (re-denaturation) with continuous fluorescence acquisition. The resulting melting curves are shown in Figure S1a.

Amplification of a series of four consecutive 1:4 dilutions of cDNA pooled from the samples with two replicates for each dilution was used to generate a standard curve with quantification cycle (Cq) values on the *Y*-axis and Log 10 of the dilution on the *X*-axis, a line fitted to the points and primer efficiency (E) calculated with the equation: E = (10^(−1/slope of standard curve) − 1) × 100. The standard curves for all the primers were within the linear dynamic range as indicated by R^2^ of the standard curves, and the Cq values of all the samples fell inside the linear dynamic range.

As negative control, ddH2O was added to the wells. No amplification signal was observed, or it was within 40–42 cycles Cq.

### 4.7. Assessment of Reference-Gene Stability

Gene expression was quantified with QuantStudio Software v1.7.2 (Applied Biosystems, Waltham, MA, USA). Cq values were determined with the Baseline-Threshold algorithm implemented in QuantStudio Software. Raw Cq values are shown in Figure S13.

Reference-gene stability and reliability were evaluated with the RefFinder web tool, which integrates the algorithms geNorm, NormFinder, BestKeeper and Delta CT.

### 4.8. Statistics

Statistical analyses were performed in GraphPad Prism v10.4 (GraphPad Software, San Diego, CA, USA).

Sample-size adequacy was assessed with G*Power v3.1.9.7 (Heinrich Heine Universität, Düsseldorf, Germany) at α = 0.05 and a test power of 98%, as described by Faul et al. [42].

## 5. Conclusions

The present study constitutes the first systematic evaluation of housekeeping-gene stability in two clinical matrices from patients with pulmonary tuberculosis—perilesional lung tissue and peripheral blood mononuclear cells. By applying four widely used ranking algorithms (geNorm, NormFinder, Delta CT and BestKeeper) and integrating their outputs in RefFinder, we show that the canonical reference genes *GAPDH*, *ACTB* and *UBC* display the lowest stability, whereas *PPIA*, *YWHAZ* and *HPRT1* reproducibly exhibit high expression stability in both specimen types. Moreover, the expression of *PPIA*, *YWHAZ* and *HPRT1* is highly correlated.

These findings broaden the methodological framework for quantitative qPCR in pulmonary TB, where accurate gene-expression normalization is essential for biomarker validation, treatment-efficacy assessment and the selection of pharmacological targets within host-directed therapy. This requirement is particularly salient amid the rapid integration of molecular-genetic approaches into clinical practice. Our study reinforces a core MIQE principle: reference-gene selection must be validated for each specimen type in accordance with disease context and pathology, thereby ensuring the reproducibility and reliability of qRT-PCR investigations.

Like the MIQE guidelines, the EU-CardioRNA COST Action guidelines serve as a tool for validating intermediate stages of biomarker research. The guidelines emphasize the importance of correctly selecting multiple, stably expressed reference genes, as demonstrated in our study. These guidelines help bridge the gap between research-only use and in vitro diagnostics. This is crucial for future work in the development of diagnostic and prognostic tests.

## Figures and Tables

**Table 1:** Primer characteristics.

Gene	Forward Primer	Reverse Primer	Product Size (bp)
*PPIA* ^†^	GTTTATGTGTCAGGGTGGTG	CGTATGCTTTAGGATGAAGTTCTC	103
*B2M* ^†^	GGGTTTCATCCATCCGACATTG	ACACGGCAGGCATACTCATCTTTT	161
*ACTB* ^††^	CTGGAACGGTGAAGGTGACA	AAGGGACTTCCTGTAACAATGCA	140
*GAPDH* ^††^	TGCACCACCAACTGCTTAGC	GGCATGGACTGTGGTCATGAG	87
*HPRT1* ^††^	TGACACTGGCAAAACAATGCA	GGTCCTTTTCACCAGCAAGCT	94
*RPL13A* ^††^	CCTGGAGGAGAAGAGGAAAGAGA	TTGAGGACCTCTGTGTATTTGTCAA	126
*UBC* ^††^	ATTTGGGTCGCAGTTCTTG	TGCCTTGACATTCTCGATGGT	133
*YWHAZ* ^††^	ACTTTTGGTACATTGTGGCTTCAA	CCGCCAGGACAAACCAGTAT	94

^†^ Primer sequences for *PPIA* and *B2M* were designed with Primer-BLAST web tool available at https://www.ncbi.nlm.nih.gov/tools/primer-blast/, accessed on 17 November 2025 (NLM, Bethesda, MD, USA). ^††^ Primer sequence for *UBC* was adopted from [11]. Primer sequences for *YWHAZ*, *ACTB*, *HPRT1*, *RPL13A*, and *GAPDH* were adopted from [12].

**Table 2:** Primer efficiencies and correlation coefficients (R^2^) for selected genes.

Gene Symbol	Gene Name	Slope	Efficiency, %	R^2^
*ACTB*	β-actin	−3.3863	97.4	0.988
*B2M*	β2-microglobulin	−3.5406	91.6	0.999
*GAPDH*	glyceraldehyde 3-phosphate dehydrogenase	−3.3093	100.5	0.996
*HPRT1*	hypoxanthine phosphoribosyl-transferase 1	−3.4958	93.2	0.998
*PPIA*	peptidylprolyl isomerase A	−3.3265	99.8	0.998
*RPL13A*	ribosomal protein L13a	−3.3978	96.9	0.996
*UBC*	ubiquitin C	−3.3656	98.2	0.999
*YWHAZ*	tyrosine 3-monooxygenase tryptophan 5-monooxygenase activation protein	−3.4538	94.8	0.996

**Table 3:** Descriptive statistics of reference gene expression for all examined reference gene candidates.

Genes	*ACTB*	*B2M*	*GAPDH*	*HPRT1*	*PPIA*	*RPL13A*	*UBC*	*YWHAZ*
Cq_Mean_ in tuberculomas	20.41	20.04	27.01	29.42	23.08	20.94	27.54	26.95
SD in tuberculomas	0.518	1.071	1.437	0.811	0.872	0.661	1.379	0.913
Cq_Mean_ in PBMCs	18.94	18.70	24.05	28.40	22.97	20.16	25.42	24.70
SD in PBMCs	0.534	0.662	0.612	0.849	0.682	0.656	1.055	0.773
Difference in Cq_Mean_ ^†^	1.47	1.34	2.96	1.02	0.11	0.78	2.12	2.25
*p*-value	<0.0001	<0.0001	<0.0001	<0.0005	0.6580	0.0008	<0.0001	<0.0001

^†^—Difference in Cq_Mean_ given as Cq_Mean_ of group “tuberculomas”—Cq_Mean_ in group “PBMCs”.

**Table 4:** BestKeeper ranking of candidate reference genes in the “tuberculomas” group. Genes are ordered by increasing standard deviation of Cq values.

Genes	CV	Standard Deviation	Correlation Coefficient	*p*-Value
*ACTB*	1.78	0.36	0.777	0.001
*RPL13A*	2.39	0.5	0.542	0.011
*HPRT1*	2.11	0.62	0.863	0.001
*PPIA*	3.04	0.7	0.884	0.001
*YWHAZ*	2.65	0.71	0.893	0.001
*B2M*	4.28	0.86	0.855	0.001
*UBC*	4.1	1.13	0.759	0.001
*GAPDH*	4.37	1.18	0.513	0.017

**Table 5:** BestKeeper ranking of candidate reference genes in the “PBMCs” group. Genes are ordered by increasing standard deviation of Cq values.

Genes	CV	Standard Deviation	Correlation Coefficient	*p*-Value
*ACTB*	2.3	0.44	0.683	0.003
*B2M*	2.43	0.45	0.873	0.001
*RPL13A*	2.36	0.48	0.723	0.001
*GAPDH*	2.05	0.49	0.678	0.003
*PPIA*	2.26	0.52	0.895	0.001
*HPRT1*	2.25	0.64	0.853	0.001
*YWHAZ*	2.62	0.65	0.961	0.001
*UBC*	3.34	0.85	0.857	0.001

**Table 6:** Integrative table with geNorm, NormFinder and Delta CT rankings for analyzed genes from groups “tuberculomas” and “PBMCs”.

Sample Group	Rank	geNorm		NormFinder		Delta CT	
		Gene	M Value	Gene	Stability Value	Gene	Stability Value
Tuberculomas	1	*HPRT1/PPIA*	0.358	*YWHAZ*	0.372	*PPIA*	0.77
	2			*HPRT1*	0.403	*HPRT1*	0.78
	3	*ACTB*	0.499	*PPIA*	0.416	*YWHAZ*	0.82
	4	*RPL13A*	0.533	*ACTB*	0.447	*ACTB*	0.84
	5	*YWHAZ*	0.574	*B2M*	0.646	*B2M*	0.93
	6	*B2M*	0.622	*RPL13A*	0.740	*RPL13A*	0.95
	7	*UBC*	0.808	*UBC*	1.016	*UBC*	1.22
	8	*GAPDH*	0.971	*GAPDH*	1.348	*GAPDH*	1.46
PBMCs	1	*HPRT1/PPIA*	0.251	*YWHAZ*	0.209	*PPIA*	0.52
	2			*PPIA*	0.283	*YWHAZ*	0.53
	3	*RPL13A*	0.302	*B2M*	0.335	*B2M*	0.58
	4	*YWHAZ*	0.383	*HPRT1*	0.486	*HPRT1*	0.62
	5	*B2M*	0.459	*RPL13A*	0.504	*RPL13A*	0.64
	6	*ACTB*	0.533	*ACTB*	0.508	*ACTB*	0.67
	7	*GAPDH*	0.576	*GAPDH*	0.533	*GAPDH*	0.68
	8	*UBC*	0.630	*UBC*	0.689	*UBC*	0.79

**Table 7:** Integrative table with BestKeeper and RefFinder rankings for analyzed genes from groups “tuberculomas” and “PBMCs”.

Sample Group	Rank	BestKeeper						RefFinder	
		Gene	SD	Gene	CV	Gene	Correlation Coefficient	Gene	Geomean
Tuberculomas	1	*ACTB*	0.36	*ACTB*	1.78	*YWHAZ*	0.893	*PPIA*	1.86
	2	*RPL13A*	0.5	*HPRT1*	2.11	*PPIA*	0.884	*HPRT1*	1.86
	3	*HPRT1*	0.62	*RPL13A*	2.39	*HPRT1*	0.863	*ACTB*	2.63
	4	*PPIA*	0.7	*YWHAZ*	2.65	*B2M*	0.855	*YWHAZ*	2.94
	5	*YWHAZ*	0.71	*PPIA*	3.04	*ACTB*	0.777	*RPL13A*	4.12
	6	*B2M*	0.86	*UBC*	4.1	*UBC*	0.759	*B2M*	5.48
	7	*UBC*	1.13	*B2M*	4.28	*RPL13A*	0.542	*UBC*	7.00
	8	*GAPDH*	1.18	*GAPDH*	4.37	*GAPDH*	0.513	*GAPDH*	8.00
PBMCs	1	*ACTB*	0.44	*GAPDH*	2.05	*YWHAZ*	0.961	*PPIA*	1.78
	2	*B2M*	0.45	*HPRT1*	2.25	*PPIA*	0.895	*YWHAZ*	2.74
	3	*RPL13A*	0.48	*PPIA*	2.26	*B2M*	0.873	*B2M*	3.08
	4	*GAPDH*	0.49	*ACTB*	2.3	*UBC*	0.857	*HPRT1*	3.13
	5	*PPIA*	0.52	*RPL13A*	2.36	*HPRT1*	0.853	*ACTB*	3.83
	6	*HPRT1*	0.64	*B2M*	2.43	*RPL13A*	0.723	*RPL13A*	3.87
	7	*YWHAZ*	0.65	*YWHAZ*	2.62	*ACTB*	0.683	*GAPDH*	6.09
	8	*UBC*	0.85	*UBC*	3.34	*GAPDH*	0.678	*UBC*	8.00

**Table 8:** Patient characteristics.

**Tuberculomas**	**Categories**	***n***	**Percent, %**
Gender	M	10	48
	F	11	52
Therapy duration, months	2–6	6	29
	7–12	4	19
	>12	11	52
**PBMCs**	**Categories**	***n***	**Percent, %**
Gender	M	9	53
	F	8	47
Therapy duration, months	2–6	8	47
	7–12	7	41
	>12	2	12

## Data Availability

The original contributions presented in this study are included in the article/Supplementary Material. Further inquiries can be directed to the corresponding author.

## Supplementary Materials

The following supporting information can be downloaded at https://www.mdpi.com/article/10.3390/ijms262211219/s1.

## Abbreviations

The following abbreviations are used in this manuscript:

PBMCs	peripheral blood mononuclear cells
*ACTB*	β-actin
*B2M*	β2-microglobulin
*GAPDH*	glyceraldehyde 3-phosphate dehydrogenase
*HPRT1*	hypoxanthine phosphoribosyl-transferase 1
*PPIA*	peptidylprolyl isomerase A
*RPL13A*	ribosomal protein L13a
*UBC*	ubiquitin C
*YWHAZ*	tyrosine 3-monooxygenase tryptophan 5-monooxygenase activation protein
