# Supplementary material for: Validation of Housekeeping Genes for Normalizing RNA Expression in Real-Time PCR in Tuberculomas and Peripheral Blood Mononuclear Cells for Pulmonary Tuberculosis Patients
Source: Int J Mol Sci. 2025 Nov 20;26(22):11219. doi: 10.3390/ijms262211219 (PMC12653038; doi:10.3390/ijms262211219)
Supplement: Supplementary file 1 [file ijms-26-11219-s001.zip › ijms-3929189-supplementary.pdf]

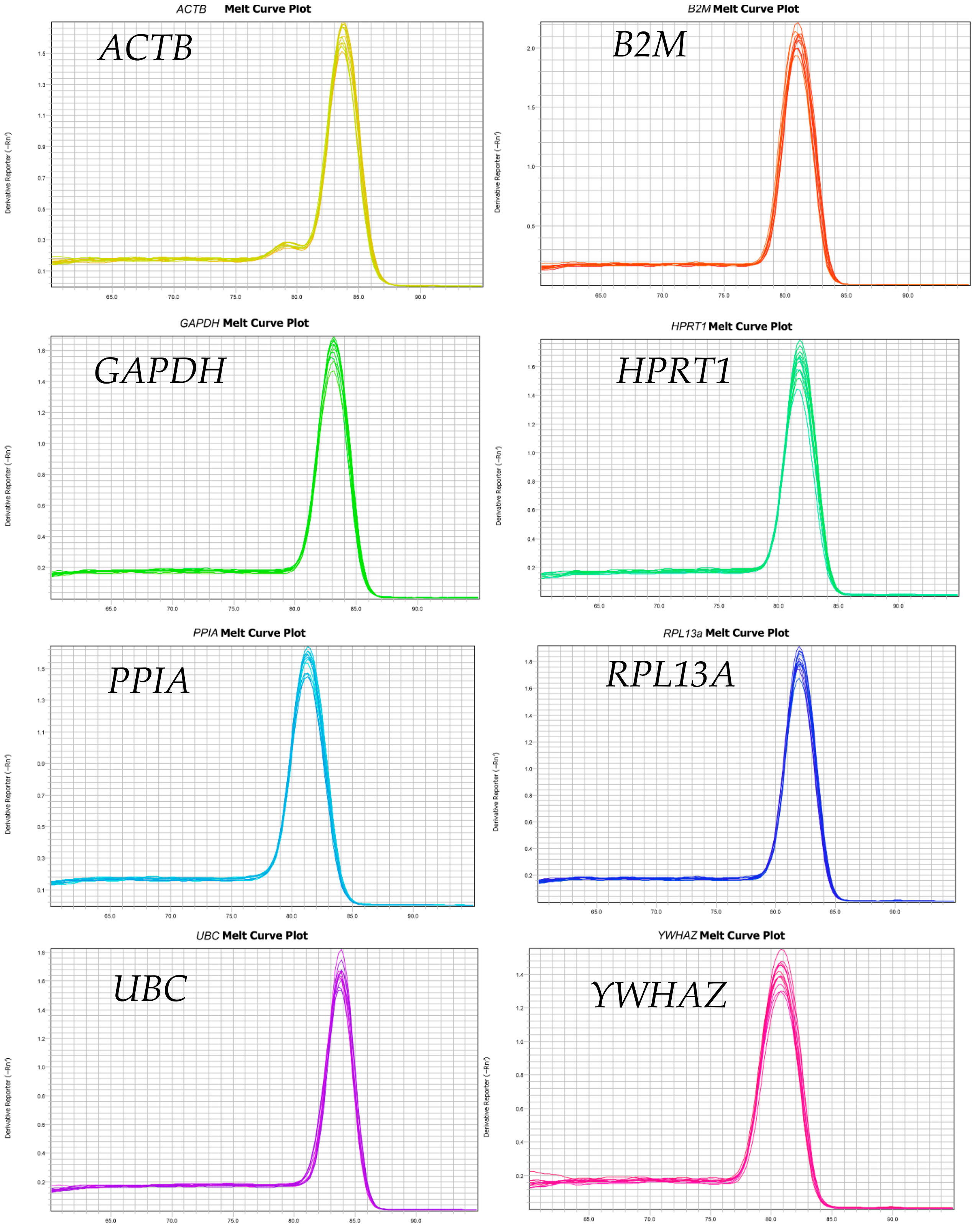

Figure S1a. Primer melting curves. Y-axis represents the derivative reporter (ΔRn) while x-axis represents the temperature (°C). For each primer set a melting curve was obtained and a single peak was detected, confirming primer specificity.

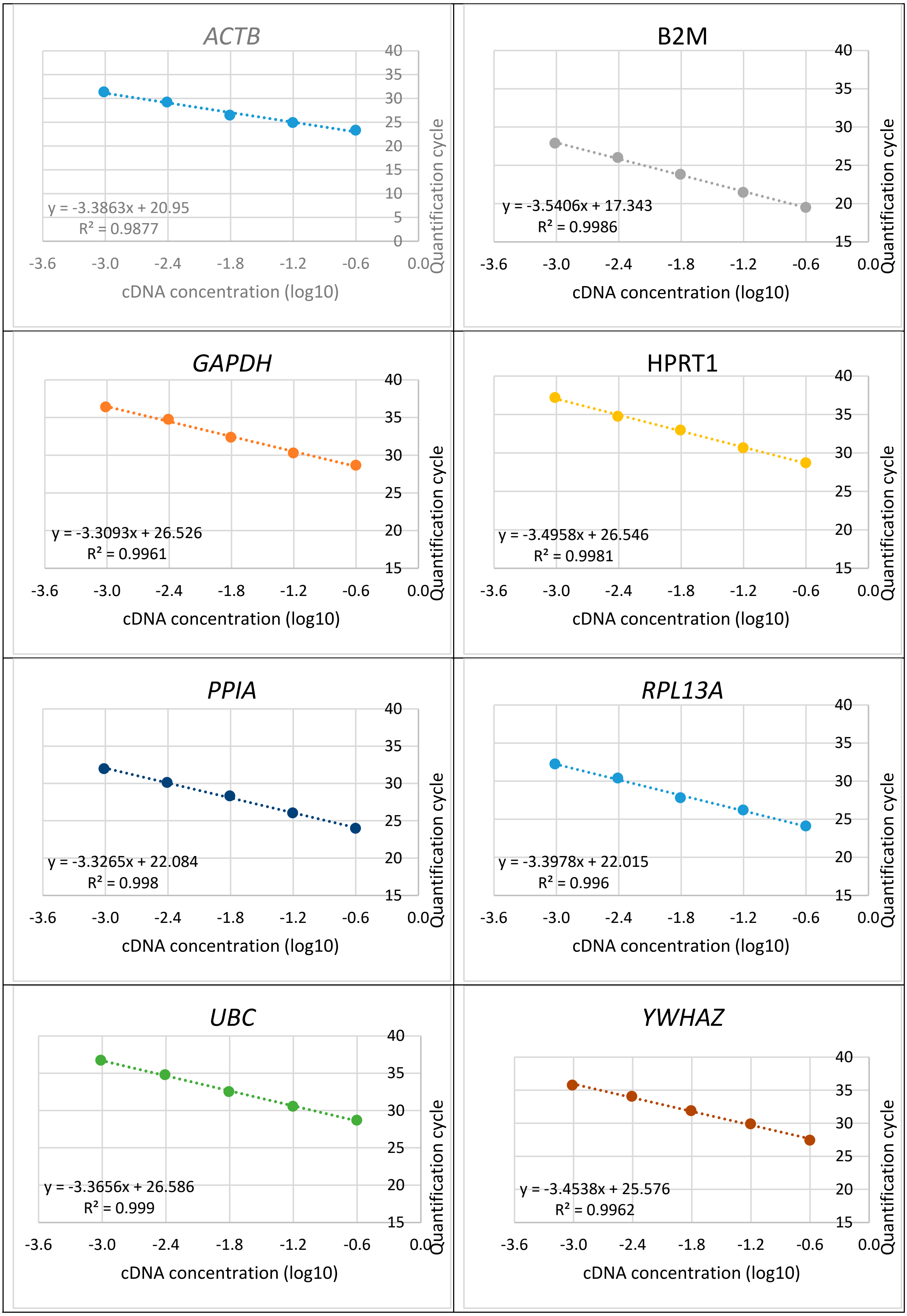

Figure S1b. Standard curve for the qRT- PCR. The X-axis represents log_{10} of the matrix concentration, and the Y-axis represents the quantification cycle value (Cq). Standard curves for the qRT- PCR represented to Figure S1b and demonstrated slopes from 3.32 (for *PPIA*) to 3.54 (for *YWHAZ*).

Table S1. Raw data from BestKeeper for the "tuberculomas" group.

	ACTB	*B2M*	*GAPDH*	*HPRT1*	*PPIA*	*RPL13A*	*UBC*	*YWHAZ*
n	21	21	21	21	21	21	21	21
geo Mean [CP]	20.4	20.01	26.97	29.41	23.07	20.93	27.51	26.94
AR Mean [CP]	20.41	20.04	27.01	29.42	23.08	20.94	27.54	26.95
min [CP]	19.67	18.45	24.89	28.39	21.63	20	24.17	25.21
max [CP]	21.61	22.25	29.88	31.56	24.96	22.47	29.37	28.76
stddev [+/- CP]	0.36	0.86	1.18	0.62	0.7	0.5	1.13	0.71
CV [% CP]	1.78	4.28	4.37	2.11	3.04	2.39	4.1	2.65
min [x-fold]	-1.66	-2.95	-4.21	-2.03	-2.71	-1.92	-10.14	-3.3
max [x-fold]	2.31	4.72	7.54	4.43	3.7	2.89	3.64	3.53
stddev [+/- x-fold]	1.29	1.81	2.27	1.54	1.63	1.42	2.19	1.64
coeff, ofcorr, [r]	0.777	0.855	0.513	0.863	0.884	0.542	0.759	0.893
p-value	0.001	0.001	0.017	0.001	0.001	0.011	0.001	0.001

Table S2. Raw data from BestKeeper for the "PBMCs" group.

	ACTB	*B2M*	*GAPDH*	*HPRT1*	*PPIA*	*RPL13A*	*UBC*	*YWHAZ*
n	17	17	17	17	17	17	17	17
geo Mean [CP]	18.93	18.69	24.04	28.38	22.96	20.15	25.4	24.69
AR Mean [CP]	18.94	18.7	24.05	28.4	22.97	20.16	25.42	24.7
min [CP]	17.72	17.57	23.05	27.33	21.97	19.37	24.14	23.45
max [CP]	19.72	20.43	25.03	30.15	24.32	21.98	27.77	26.12
stddev [+/- CP]	0.44	0.45	0.49	0.64	0.52	0.48	0.85	0.65
CV [% CP]	2.3	2.43	2.05	2.25	2.26	2.36	3.34	2.62
min [x-fold]	-2.32	-2.18	-1.99	-2.08	-1.98	-1.71	-2.39	-2.37
max [x-fold]	1.72	3.34	1.98	3.41	2.58	3.55	5.15	2.69
stddev [+/- x-fold]	1.35	1.37	1.41	1.56	1.43	1.39	1.8	1.57
coeff.ofcorr. [r]	0.683	0.873	0.678	0.853	0.895	0.723	0.857	0.961
p-value	0.003	0.001	0.003	0.001	0.001	0.001	0.001	0.001

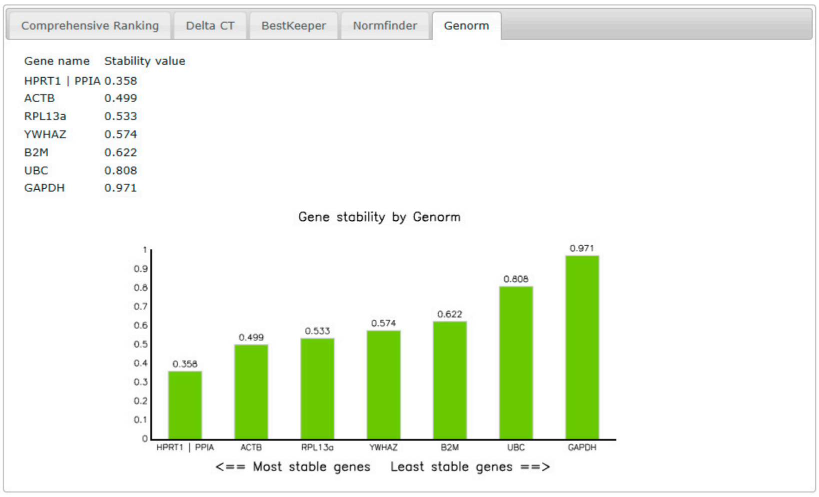

Figure S2. geNorm gene rankings for the "tuberculomas" group from the RefFinder web tool [20].

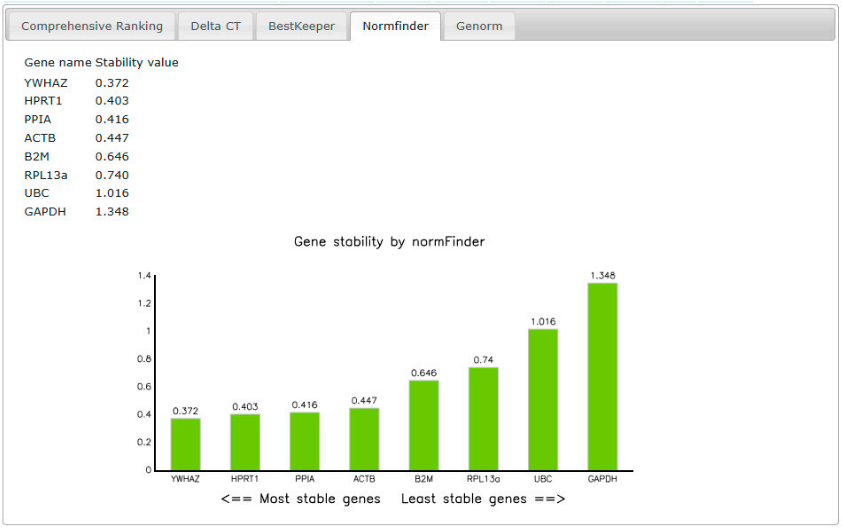

Figure S3. NormFinder gene rankings for the "tuberculomas" group from the RefFinder web tool [20].

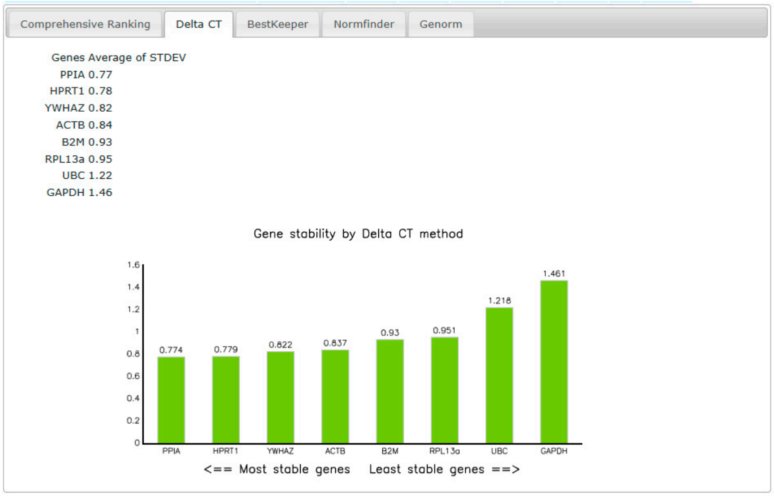

Figure S4. Delta CT gene rankings for the "tuberculomas" group from the RefFinder web tool [20].

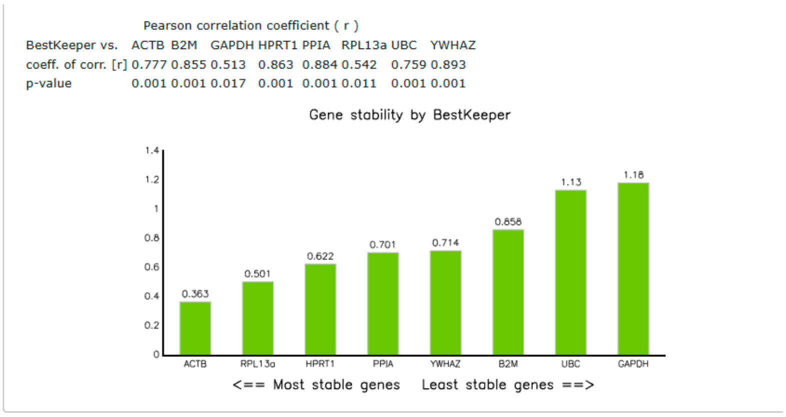

Figure S5. BestKeeper gene rankings for the "tuberculomas" group from the RefFinder web tool [20].

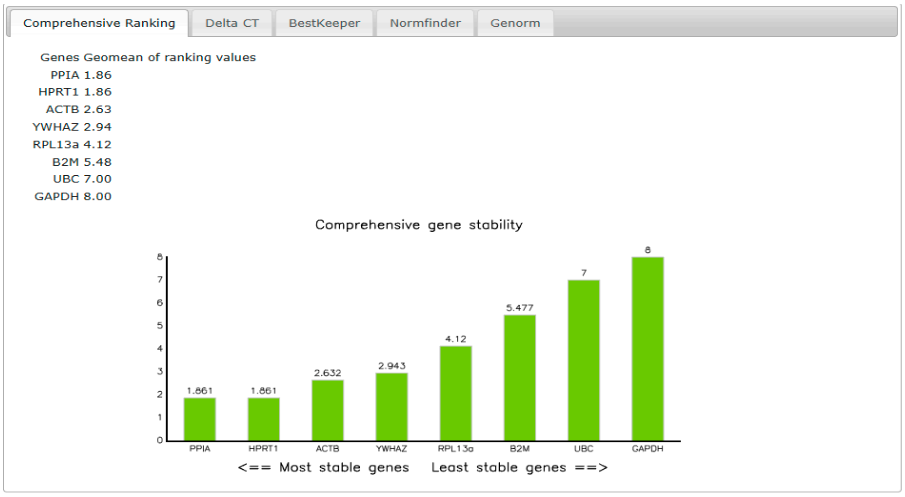

Figure S6. RefFinder comprehensive gene rankings for the "tuberculomas" group from the RefFinder web tool [20].

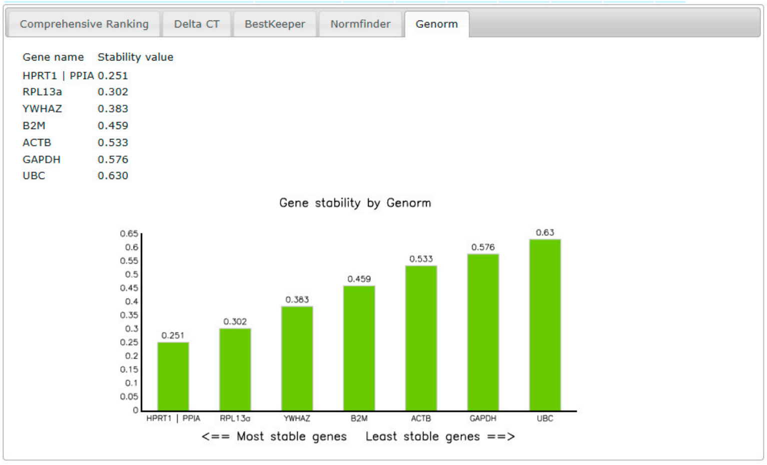

Figure S7. geNorm gene rankings for the "PMBCs" group from the RefFinder web tool [20].

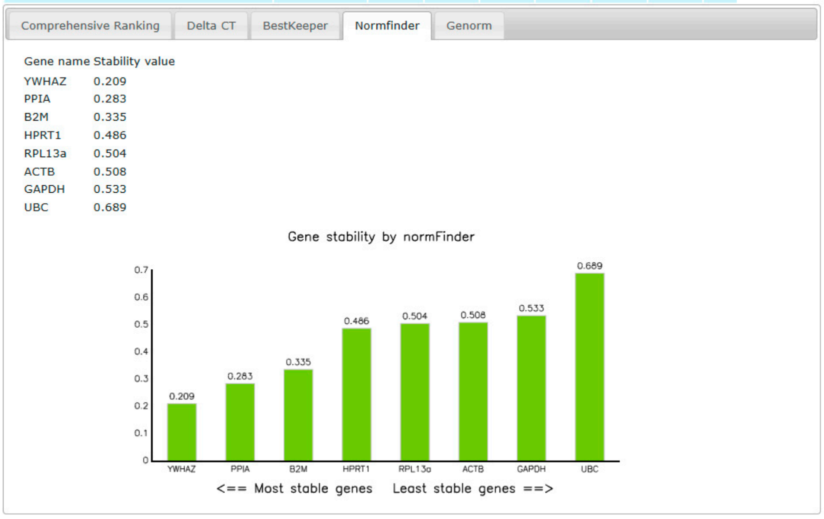

Figure S8. NormFinder gene rankings for the "PMBCs" group from the RefFinder web tool [20].

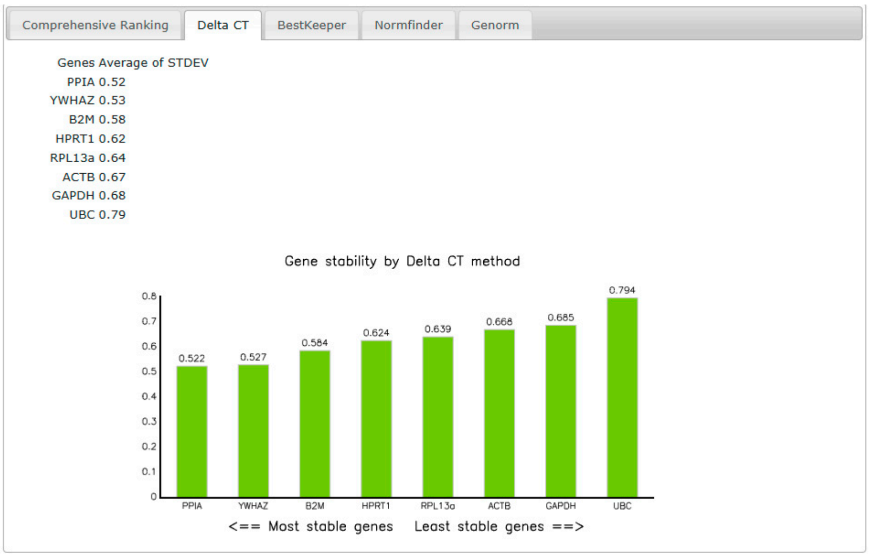

Figure S9. Delta CT gene rankings for the "PMBCs" group from the RefFinder web tool [20].

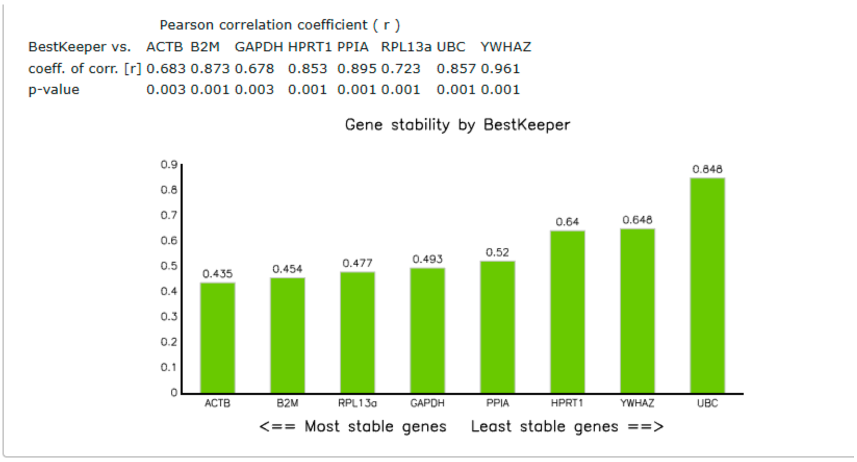

Figure S10. BestKeeper gene rankings for the “PMBCs” group from the RefFinder web tool [20].

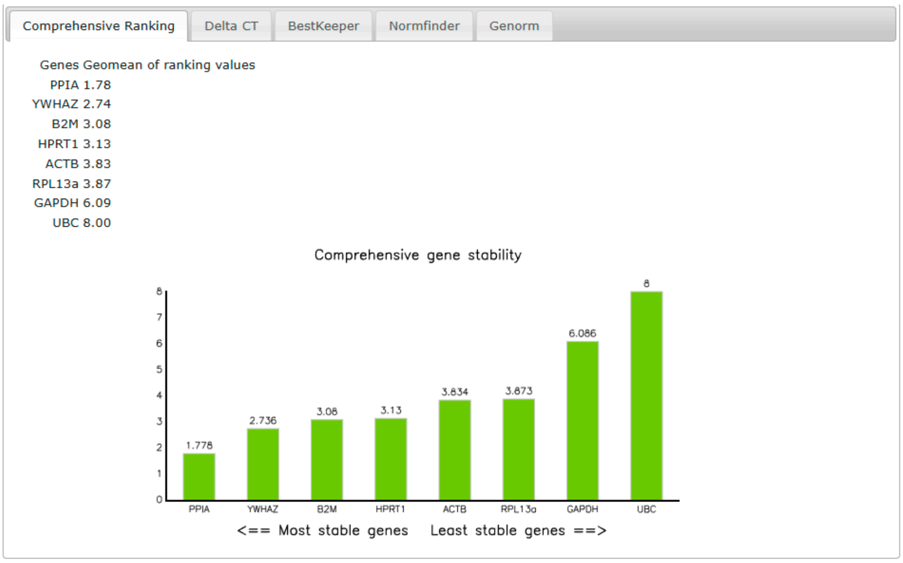

Figure S11. RefFinder comprehensive gene rankings for the “PMBCs” group from the RefFinder web tool [20].

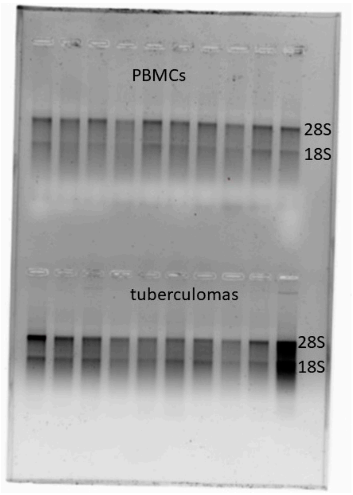

Figure S12. RNA integrity check via gel-electrophoresis. Upper row – group “PBMCs”, lower row – group “tuberculomas”.18S and 28S strands are visible.

Sample Name	Gene	EQ.Ct Mean	Gene	EQ.Ct Mean	Gene	EQ.Ct Mean	Gene	EQ.Ct Mean	Gene	EQ.Ct Mean	Gene	EQ.Ct Mean	Gene	EQ.Ct Mean	Gene	EQ.Ct Mean
Tuberculoma_01	B2M	20,803	ACTB	20,873	GAPDH	29,436	HPRT1	29,824	PPIA	23,209	RPL13a	20,962	UBC	29,374	YWHAZ	27,160
Tuberculoma_02	B2M	21,931	ACTB	20,445	GAPDH	26,782	HPRT1	29,799	PPIA	24,085	RPL13a	21,219	UBC	27,076	YWHAZ	27,015
Tuberculoma_03	B2M	20,062	ACTB	20,285	GAPDH	29,883	HPRT1	28,485	PPIA	22,674	RPL13a	20,149	UBC	28,188	YWHAZ	26,144
Tuberculoma_04	B2M	20,993	ACTB	21,613	GAPDH	26,409	HPRT1	30,450	PPIA	24,088	RPL13a	22,466	UBC	26,784	YWHAZ	26,963
Tuberculoma_05	B2M	19,019	ACTB	20,033	GAPDH	26,249	HPRT1	28,587	PPIA	21,629	RPL13a	20,043	UBC	26,024	YWHAZ	25,420
Tuberculoma_06	B2M	19,815	ACTB	20,022	GAPDH	24,993	HPRT1	29,234	PPIA	22,683	RPL13a	21,399	UBC	25,381	YWHAZ	25,906
Tuberculoma_07	B2M	19,543	ACTB	20,832	GAPDH	28,105	HPRT1	29,340	PPIA	22,814	RPL13a	20,868	UBC	28,949	YWHAZ	27,277
Tuberculoma_08	B2M	18,446	ACTB	19,818	GAPDH	26,147	HPRT1	28,691	PPIA	22,028	RPL13a	19,995	UBC	26,237	YWHAZ	26,105
Tuberculoma_09	B2M	18,973	ACTB	19,674	GAPDH	24,894	HPRT1	28,390	PPIA	21,972	RPL13a	20,678	UBC	24,167	YWHAZ	25,214
Tuberculoma_10	B2M	18,770	ACTB	20,418	GAPDH	27,182	HPRT1	28,729	PPIA	22,415	RPL13a	20,761	UBC	27,584	YWHAZ	26,900
Tuberculoma_11	B2M	18,579	ACTB	20,333	GAPDH	25,967	HPRT1	28,905	PPIA	22,560	RPL13a	20,418	UBC	26,210	YWHAZ	26,519
Tuberculoma_12	B2M	19,349	ACTB	19,913	GAPDH	27,834	HPRT1	28,723	PPIA	22,420	RPL13a	20,430	UBC	28,322	YWHAZ	26,557
Tuberculoma_13	B2M	19,879	ACTB	20,447	GAPDH	27,862	HPRT1	29,534	PPIA	22,992	RPL13a	20,563	UBC	28,374	YWHAZ	27,595
Tuberculoma_14	B2M	20,118	ACTB	20,408	GAPDH	28,893	HPRT1	29,458	PPIA	23,174	RPL13a	20,668	UBC	29,209	YWHAZ	27,725
Tuberculoma_15	B2M	19,702	ACTB	20,141	GAPDH	27,922	HPRT1	29,317	PPIA	23,008	RPL13a	20,953	UBC	28,871	YWHAZ	27,369
Tuberculoma_16	B2M	19,669	ACTB	19,674	GAPDH	25,290	HPRT1	28,733	PPIA	22,433	RPL13a	21,413	UBC	26,888	YWHAZ	26,308
Tuberculoma_17	B2M	19,672	ACTB	20,404	GAPDH	25,011	HPRT1	29,416	PPIA	23,600	RPL13a	21,465	UBC	27,249	YWHAZ	27,359
Tuberculoma_18	B2M	21,401	ACTB	21,576	GAPDH	25,925	HPRT1	30,636	PPIA	24,955	RPL13a	22,352	UBC	27,950	YWHAZ	28,759
Tuberculoma_19	B2M	22,247	ACTB	20,650	GAPDH	27,709	HPRT1	31,561	PPIA	24,531	RPL13a	21,530	UBC	28,281	YWHAZ	28,429
Tuberculoma_20	B2M	20,704	ACTB	20,489	GAPDH	27,290	HPRT1	29,903	PPIA	23,677	RPL13a	20,625	UBC	28,354	YWHAZ	27,337
Tuberculoma_21	B2M	21,069	ACTB	20,580	GAPDH	27,328	HPRT1	30,168	PPIA	23,787	RPL13a	20,877	UBC	28,925	YWHAZ	27,932
PBMC_01	B2M	17,968	ACTB	18,281	GAPDH	23,406	HPRT1	27,453	PPIA	22,064	RPL13a	19,374	UBC	24,183	YWHAZ	23,573
PBMC_02	B2M	19,567	ACTB	18,710	GAPDH	23,837	HPRT1	28,626	PPIA	23,277	RPL13a	20,307	UBC	26,538	YWHAZ	25,193
PBMC_03	B2M	18,330	ACTB	18,692	GAPDH	23,981	HPRT1	27,678	PPIA	22,235	RPL13a	19,930	UBC	24,257	YWHAZ	24,067
PBMC_04	B2M	20,432	ACTB	19,717	GAPDH	24,958	HPRT1	29,908	PPIA	23,928	RPL13a	20,788	UBC	27,766	YWHAZ	26,123
PBMC_05	B2M	19,353	ACTB	19,081	GAPDH	24,095	HPRT1	28,508	PPIA	22,891	RPL13a	19,871	UBC	26,126	YWHAZ	24,917
PBMC_06	B2M	18,320	ACTB	18,462	GAPDH	23,423	HPRT1	27,891	PPIA	22,413	RPL13a	19,381	UBC	24,887	YWHAZ	24,053
PBMC_07	B2M	18,498	ACTB	19,454	GAPDH	24,886	HPRT1	27,920	PPIA	22,888	RPL13a	19,906	UBC	25,682	YWHAZ	24,680
PBMC_08	B2M	18,613	ACTB	19,430	GAPDH	24,012	HPRT1	28,728	PPIA	23,294	RPL13a	20,397	UBC	24,646	YWHAZ	24,196
PBMC_09	B2M	18,006	ACTB	18,719	GAPDH	23,487	HPRT1	27,325	PPIA	21,969	RPL13a	19,396	UBC	24,143	YWHAZ	23,447
PBMC_10	B2M	18,549	ACTB	19,077	GAPDH	23,967	HPRT1	27,971	PPIA	22,755	RPL13a	20,280	UBC	25,068	YWHAZ	24,339
PBMC_11	B2M	19,079	ACTB	19,657	GAPDH	23,819	HPRT1	28,599	PPIA	23,175	RPL13a	20,451	UBC	25,019	YWHAZ	24,828
PBMC_12	B2M	18,677	ACTB	19,458	GAPDH	24,765	HPRT1	28,145	PPIA	22,963	RPL13a	20,081	UBC	26,031	YWHAZ	25,190
PBMC_14	B2M	18,627	ACTB	19,192	GAPDH	25,032	HPRT1	28,382	PPIA	23,259	RPL13a	20,036	UBC	27,018	YWHAZ	25,448
PBMC_15	B2M	18,703	ACTB	18,676	GAPDH	23,674	HPRT1	30,153	PPIA	24,323	RPL13a	21,975	UBC	25,343	YWHAZ	25,641
PBMC_16	B2M	18,853	ACTB	18,552	GAPDH	23,716	HPRT1	29,691	PPIA	23,932	RPL13a	20,960	UBC	25,261	YWHAZ	25,358
PBMC_17	B2M	17,565	ACTB	17,717	GAPDH	23,050	HPRT1	27,404	PPIA	22,128	RPL13a	19,615	UBC	24,191	YWHAZ	23,773
PBMC_18	B2M	18,780	ACTB	19,085	GAPDH	24,758	HPRT1	28,349	PPIA	22,946	RPL13a	19,941	UBC	25,996	YWHAZ	25,156

Figure S13. Raw Cq (Ct) values for each gene analyzed in groups "tuberculomas" and "PBMCs".
